# P04.73. Reflexology, cardiac patients and inconsistencies in the location of the heart reflex point: an online survey

**DOI:** 10.1186/1472-6882-12-S1-P343

**Published:** 2012-06-12

**Authors:** J Jones, P Thomson, S Leslie

**Affiliations:** 1 University of Stirling, Inverness, United Kingdom; 2 Highland Heartbeat Centre, Cardiology Unit, Raigmore Hospital, Inverness, United Kingdom

## Purpose

This survey aimed to generate preliminary data, in the face of inconsistent reflexology teaching literature, about two reflexology safety and quality control issues. First, whether reflexologists consider it safe to treat people with heart disease and if so, whether the presence of cardiac disease influences the therapist’s treatment decisions. Reflexology teaching literature is contradictory on this subject, with ‘heart' disease listed as both an indication, which can benefit from treatment, and as a significant contraindication where treatment should be avoided. The second concern is one of product quality, namely the issue of inconsistency of reflex points in reflexology maps.

## Methods

An online survey invitation email was sent to all therapists in the membership database of the Association of Reflexologists. Survey questions included “do you treat clients with diagnosed heart problems”, “if you became aware of imbalances in the heart reflex area, what would you do”, “would you limit the treatment” and “would you expect the treatment to improve their heart condition? The email also contained a graphical attachment, an illustration of a blank outline of the feet with transparent bone structure. We asked respondents to mark where they placed the heart reflex point on the template.

## Results

The survey has shown that reflexologists beliefs and practises mirror the inconsistencies in the reflexology teaching literature, with almost a third of the respondents expressing concerns about treating cardiac patients and over half stating that they would limit or modify the treatment as a result. Furthermore, a signficant number of respondents demonstrated a marked level of inconsistency in heart reflex point placement on the foot template.

## Conclusion

The survey findings demonstrate a lack of professional consistency and clarity regarding the suitability of reflexology for cardiac patients, marked inconsistencies in the heart reflex point placement and inconsistency in reflexologists treatment decisions for perceived heart imbalances.

